# Evaluation of Delayed Bleeding Prevention and Sustained Closure Using the Reopenable Clip‐Over‐the‐Line Method for Gastric Endoscopic Submucosal Dissection

**DOI:** 10.1002/deo2.70302

**Published:** 2026-02-26

**Authors:** Gen Kitahara, Takuya Wada, Kenta Murotani, Kusutaro Doi, Toshiki Horii, Tomohiro Betto, Akinori Watanabe, Kenji Ishido, Hisatomo Ikehara, Chika Kusano

**Affiliations:** ^1^ Department of Gastroenterology Kitasato University School of Medicine Kanagawa Japan; ^2^ School of Medical Technology Kurume University Fukuoka Japan; ^3^ Biostatistics Center Kurume University Fukuoka Japan

**Keywords:** bleeding, endoscopic submucosal dissection, gastric neoplasms, sutures, wound closure techniques

## Abstract

**Objectives:**

Delayed bleeding is a major complication of gastric endoscopic submucosal dissection (ESD), particularly in high‐risk patients. The reopenable clip‐over‐the‐line method (ROLM) is a novel closure technique, but its durability and preventive effects remain unclear. We evaluated the impact of ROLM on delayed bleeding and sustained closure after gastric ESD in high‐risk patients.

**Methods:**

We retrospectively reviewed gastric ESD cases from April 2021 to December 2024, including only patients with a BEST‐J score ≥3, and compared ROLM and non‐ROLM groups. In the ROLM group, mucosal closure was confirmed endoscopically on postoperative day 1 (POD1), and clip retention was assessed at 2 months. The primary outcome was delayed bleeding within 28 days. Secondary outcomes included sustained closure on POD1 and clip retention at 2 months.

**Results:**

The ROLM group included 21 lesions in 15 patients, and the non‐ROLM group included 93 lesions in 72 patients. Delayed bleeding occurred in 0/15 ROLM patients (0%) versus 17/72 non‐ROLM patients (23.6%) (*p* = 0.036). Multivariable models using Firth logistic regression consistently showed a reduced odds ratio (≈ 0.10), although not statistically significant. All ROLM lesions achieved sustained closure on POD1 (100%). At 2 months, 80.9% retained clips.

**Conclusion:**

In high‐risk gastric ESD patients, ROLM was associated with no delayed bleeding and achieved complete POD1 closure with frequent 2‐month clip retention, suggesting a potential for a promising prophylactic strategy.

## Graphical abstract

ROLM achieves complete closure of post‐ESD mucosal defects and is associated with no delayed bleeding in high‐risk patients. Complete closure is confirmed in 100% of lesions on postoperative day 1, and clip retention at 2 months remains high, suggesting the potential for durable performance of ROLM.

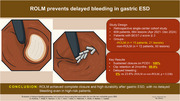

## 1 Introduction

Endoscopic submucosal dissection (ESD) enables less invasive en bloc resection of superficial gastrointestinal neoplasms, preserving organ function and postoperative quality of life. En bloc resection facilitates curative treatment through complete removal and accurate histopathological evaluation. Compared with surgery, ESD is also associated with shorter hospital stays and improved cost efficiency [1]. These advantages have led to its widespread adoption in clinical practice [2, 3, 4].

An earlier report described post‐ESD bleeding rates of 0%–15.6% [5], whereas recent reports, including multicenter retrospective studies, have reported rates of 2.7%–8.5% [6, 7, 8]. Although relatively infrequent, delayed bleeding remains one of the most clinically significant adverse events after gastric ESD because it often requires emergency intervention and can prolong hospitalization. Particularly, patients receiving antithrombotic agents or those with multiple comorbidities are at a substantially increased risk. The BEST‐J score, a large‐scale Japanese multicenter risk stratification tool, reported bleeding rates of 11.4% in high‐risk patients (score 3–4) and 29.7% in very high‐risk patients (score ≥5) [9]. Therefore, effective preventive strategies are needed for high‐risk patients undergoing ESD.

To address this challenge, several endoscopic defect closure techniques have been investigated to prevent delayed bleeding, including endoloop and endoclips closure [10], endoclip‐and‐line technique [11], endoscopic O‐ring ligation [12], and endoscopic hand suturing [13]. The reopenable clip‐over‐the‐line method (ROLM) has recently gained attention because it enables secure mucosal closure using standard devices [14]. Preliminary studies have reported high technical success and complete closure, even for large defects or difficult locations. In particular, a multicenter propensity score‐matched study by Sugimoto et al. reported that ROLM significantly lowered bleeding rates even among patients with BEST‐J scores ≥3 [15]. However, systematic endoscopic confirmation of closure after ROLM was not performed, and durability over time was not assessed. At our institution, almost all patients undergo routine second‐look endoscopy on postoperative day 1 (POD1) as well as scheduled endoscopic follow‐up at 2 months. This unique practice allows direct visualization of the early postoperative closure status and its subsequent course.

Therefore, we evaluated delayed bleeding and closure durability after ROLM in high‐risk patients.

## 2 Methods

### 2.1 Study Design and Setting

This was a retrospective single‐center cohort study at Kitasato University Hospital, including consecutive patients who underwent gastric ESD between April 2021 and December 2024.

### 2.2 Patient Eligibility

**FIGURE 1:**
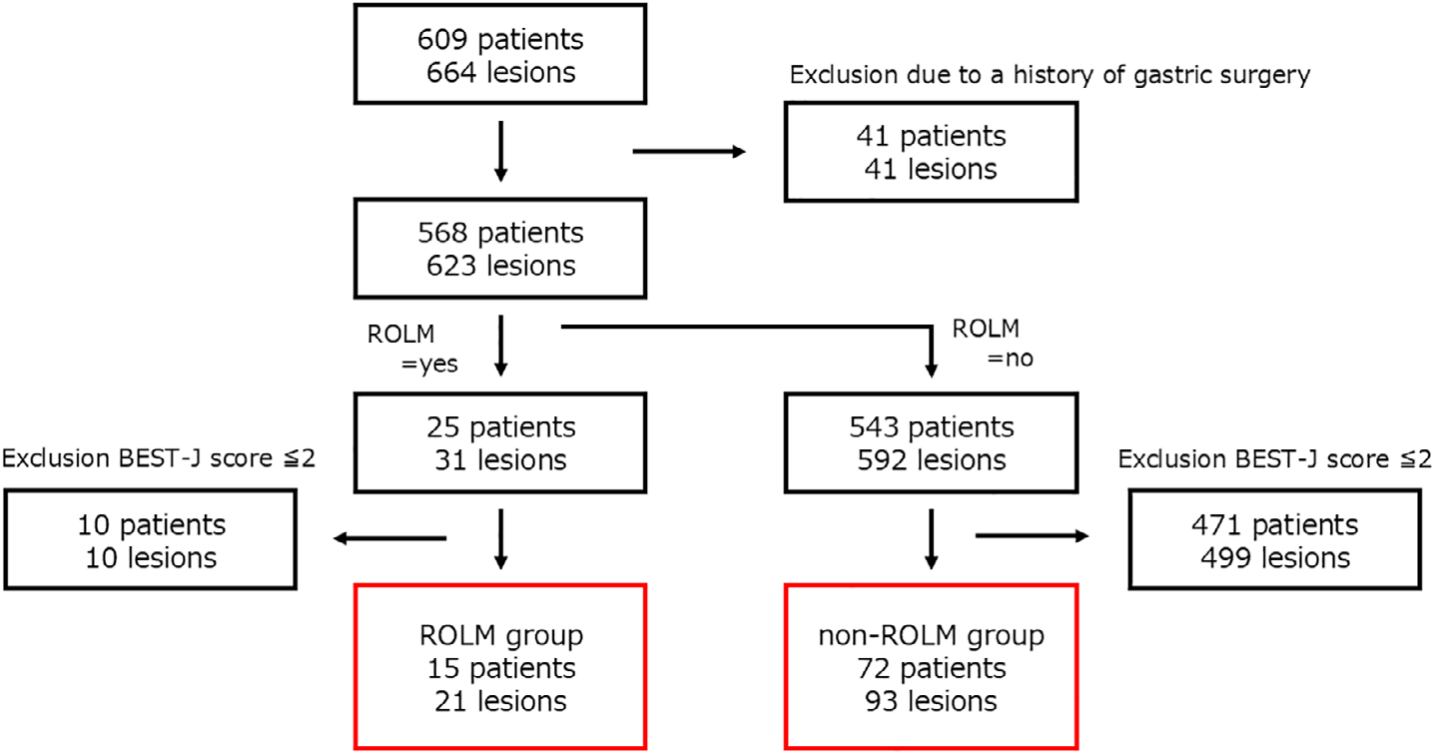
Schema of patient enrollment protocol.

All patients who underwent ESD for early gastric neoplasms during the study period were retrospectively reviewed. Patients with prior gastric surgery were excluded to avoid anatomical and healing variability. To ensure a homogeneous bleeding risk profile, only patients with a BEST‐J score of ≥3 (high/very high‐risk) were included. Additionally, patients were categorized into two groups based on the closure technique employed: the ROLM group, in which mucosal defects were closed using ROLM, and the non‐ROLM group, in which no defect closure technique but standard prophylactic management was applied (Figure 1).

In principle, ROLM was attempted in all eligible high‐risk patients after its introduction at our institution in June 2023. However, operational constraints, including endoscopist availability and procedural scheduling, occasionally precluded its use, and all cases treated before its adoption were assigned to the non‐ROLM group.

Patient‐level clinicopathological variables included sex, age, Eastern Cooperative Oncology Group performance status (ECOG‐PS), American Society of Anesthesiologists physical status classification (ASA) [16], Charlson Comorbidity Index (CCI) [17], use of antiplatelet agents or anticoagulant agents, hemodialysis, multiple lesions, BEST‐J score, and degree of gastric atrophy (closed/open/none).

Lesion‐level clinicopathological variables included location 1 (upper/middle/lower third), location 2 (lesser curvature/greater curvature/anterior wall/posterior wall), macroscopic type (elevated/depressed/mixed), depth of tumor (M, mucosal invasion; SM1, minute submucosal invasion [<500 µm below the muscularis mucosae]; SM2, massive submucosal invasion [≥500 µm below the muscularis mucosae]), ulcer scar, histologic type (intestinal/diffuse), lymphatic and venous invasion, specimen size, and ESD procedure time.

### 2.3 Endoscopic Procedures

**FIGURE 2:**
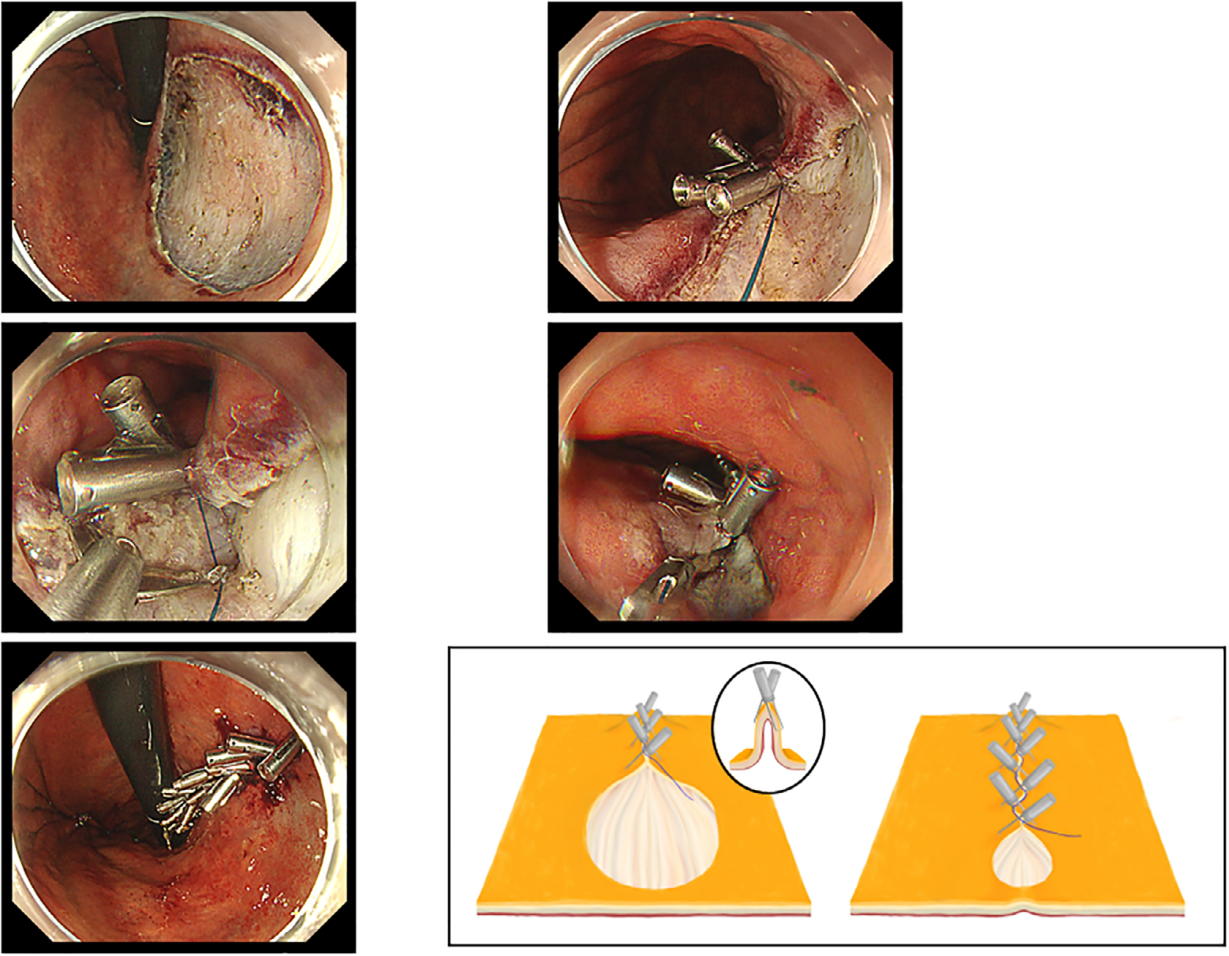
Illustration of the reopenable clip‐over‐the‐line method (ROLM).

All ESD procedures were performed using a therapeutic endoscope (GIF‐H290T, GIF‐Q260J, or GIF‐2TQ260M; Olympus, Tokyo, Japan) equipped with an insulated‐tip knife (IT Knife 2; Olympus, Tokyo, Japan). An electrosurgical generator (VIO 3; Erbe Elektromedizin GmbH, Germany) was used with Endocut I (effect 3, duration 3, interval 3) and Swift coagulation (effect 5.0). Procedures were performed under conscious sedation. In the ROLM group, mucosal defect closure was performed immediately after ESD, using standard reopenable clips and a nylon line to bring mucosal edges together following the Z‐pattern closure technique (Figure 2). In the non‐ROLM group, standard prophylactic hemostasis was performed according to institutional practice, including coagulation of exposed vessels and/or placement of standard endoclips when necessary. In patients receiving antithrombotic agents, drug withdrawal was managed in accordance with the guidelines issued by the Japan Gastroenterological Endoscopy Society in 2017 [18].

Vonoprazan (20 mg/day) was administered from the day before ESD through the 2‐month follow‐up.

Routine second‐look endoscopy on POD1 is part of our institutional post‐ESD management protocol for early adverse event detection and ulcer assessment, regardless of defect closure. Accordingly, POD1 endoscopy was performed in both groups, and closure status in the ROLM group (complete vs. non‐complete) and ulcer‐bed appearance in the non‐ROLM group were assessed. Follow‐up endoscopy at 2 months was also scheduled for all patients to confirm ulcer healing and detect synchronous lesions.

### 2.4 Histopathological Evaluation

Histopathological evaluation was conducted in accordance with the Japanese Classification of Gastric Carcinoma and the World Health Organization classification [19]. The tumor size, depth of invasion, presence of ulcerative changes, lymphatic and vascular invasion, and involvement of the lateral and vertical margins were assessed.

### 2.5 Definitions

Delayed bleeding was defined as clinical evidence of bleeding within 28 days after ESD requiring blood transfusion or urgent endoscopic intervention, and/or a hemoglobin decrease of >2 g/dL.

Perforation was defined as endoscopic visualization of intra‐abdominal organs or fat tissue during ESD, or free air on post‐procedure radiography or computed tomography.

Complete closure was defined as full coverage of the mucosal defect with no visible ulcer‐bed. Sustained closure was defined as complete closure confirmed on POD1 s‐look endoscopy.

### 2.6 Outcomes

The primary endpoint was delayed bleeding within 28 days after ESD.

Secondary endpoints were the sustained closure of the mucosal defect on POD1 and clip retention at 2 months as exploratory indicators of the durability of mucosal apposition. Clip retention was visually classified as complete retention (all clips retained), partial retention (at least one clip retained), or complete loss (no clips retained). All clinical outcomes were ascertained from electronic medical records, endoscopy reports, and laboratory data.

POD1 and 2‐month follow‐up findings were independently reviewed by three experienced endoscopists; discrepancies among them were resolved by majority decision.

No patient was lost to follow‐up during the observation period, and no missing data were found for any primary or secondary outcomes.

### 2.7 Statistical Analysis

Fisher's exact test was used for categorical variables and the Mann–Whitney U test for continuous variables. Kaplan–Meier analysis with log‐rank test was used to compare the time to delayed bleeding within 28 days after ESD. These analyses were conducted using EZR software (Saitama Medical Center, Jichi Medical University, Saitama, Japan).

Potential selection bias was addressed by estimating propensity scores for receiving ROLM using logistic regression, including age, sex, ECOG‐PS, ASA, CCI, use of antiplatelet and anticoagulant agents, hemodialysis, multiple lesions, atrophy, and BEST‐J score. Inverse probability of treatment weighting (IPTW) was applied to construct a weighted pseudo‐population, and covariate balance was assessed using standardized mean differences (SMD) (Table S1).

Given the small sample size and low event incidence, Firth logistic regression analysis was performed to minimize bias from ordinary logistic regression in rare‐event analysis and to evaluate the association between ROLM and delayed bleeding [20]. Covariates, including the CCI, antithrombotic agents, hemodialysis, and multiple lesions, were sequentially included in the multivariable models. Statistical significance was defined as a two‐sided p‐value of <0.05. Firth logistic regression analysis was conducted using SAS 9.4 (SAS Institute, Inc., Cary, NC).

## 3 Results

### 3.1 Patient Characteristics

Patient characteristics are shown in Table 1. A total of 87 patients were included in the analysis: 15 in the ROLM group and 72 in the non‐ROLM group. Sex, age, ECOG‐PS, ASA, CCI, use of antiplatelet or anticoagulant agents, frequency of multiple lesions, and degree of gastric mucosal atrophy did not differ significantly between the groups.

**TABLE 1:** Patient characteristics.

	ROLM *n* = 15	non‐ROLM *n* = 72	*p*
Sex (male/female)	13/2	63/9	1.000
Age, median (range), years	77 (64–87)	77 (58–89)	0.831
ECOG‐PS (0–1/2+)	13/2	69/3	0.204
ASA (1–2/3+)	6/9	28/44	1.000
CCI (0–1/2+)	3/12	20/52	0.750
Antiplatelet (yes/no)	11/4	36/36	0.154
Anticoagulant (yes/no)	5/10	36/36	0.270
HD (yes/no)	4/11	5/67	0.044
Multiple lesions (yes/no)	6/9	21/51	0.540
BEST‐J (3–4/5+)	8/7	58/14	0.043
Atrophy (closed/open/none)	1/13/1	12/59/1	0.302

Abbreviations: ASA, American Society of Anesthesiologists physical status; CCI, Charlson Comorbidity Index; ECOG‐PS, Eastern Cooperative Oncology Group performance status; HD, hemodialysis; ROLM, reopenable clip‐over‐the‐line method.

In contrast, hemodialysis was more frequent in the ROLM group than in the non‐ROLM group (4/15 [26.7%] vs. 5/72 [6.9%], *p* = 0.044). Patients in the ROLM group were also more likely to have a BEST‐J score ≥5 (7/15 [46.7%] vs. 14/72 [19.4%], *p* = 0.043).

After applying IPTW, SMDs were generally reduced, indicating improved covariate balance, although a higher BEST‐J score remained more frequent in the ROLM group (Table S1). In the IPTW‐weighted analysis, delayed bleeding showed complete separation, consistent with the unweighted analysis.

### 3.2 Lesion Characteristics

Lesion characteristics are shown in Table 2. A total of 114 lesions were analyzed: 21 in the ROLM group and 93 in the non‐ROLM group. No significant differences were observed between the groups in location, macroscopic type, depth of invasion, ulcer scarring, histology, lymphatic or venous invasion, specimen size, or ESD procedure time.

**TABLE 2:** Lesion characteristics.

	ROLM *n* = 21	non‐ROLM *n* = 93	*p*
Location1 (upper third/middle third/lower third)	0/8/13	9/39/45	0.327
Location2 (lesser curvature/greater curvature/anterior wall/posterior wall)	8/2/7/4	37/25/10/21	0.056
Macroscopic type (elevated/depressed/mixed)	14/6/1	47/41/5	0.363
Depth of tumor (M/SM1/SM2)	20/1/0	89/1/3	0.395
Ulcer scar findings (yes/no)	0/21	3/90	1.000
Histologic type (intestinal/diffuse)	21/0	92/1	1.000
Lymphatic invasion (yes/no)	0/21	1/92	1.000
Venous invasion (yes/no)	0/21	1/92	1.000
Specimen size, median (range), mm	35 (24–63)	33 (18–105)	0.269
ESD procedure time, median (range), min	32 (6–71)	44 (4–172)	0.055

Abbreviations: ESD, endoscopic submucosal dissection; ROLM, reopenable clip‐over‐the‐line method.

M, mucosal invasion; SM1, minute submucosal invasion (<500 µm below the muscularis mucosae); SM2, massive submucosal invasion (≥500 µm below the muscularis mucosae).

### 3.3 Clinical Outcomes

Clinical outcomes are shown in Table 3.

**TABLE 3:** Clinical outcomes.

(Patients)	ROLM *n* = 15	non‐ROLM *n* = 72	*p*
Delayed bleeding (yes/no)	0/15	17/55	0.036
Delayed perforation (yes/no)	0/15	1/71	1.000

(Lesions)	ROLM *n* = 21
Complete closure on POD1 (yes/no)	21/0
Clip retention at 2 months (complete retention/partial retention/complete loss)	4/13/4

Abbreviations: POD1, postoperative day 1; ROLM, reopenable clip‐over‐the‐line method.

**FIGURE 3:**
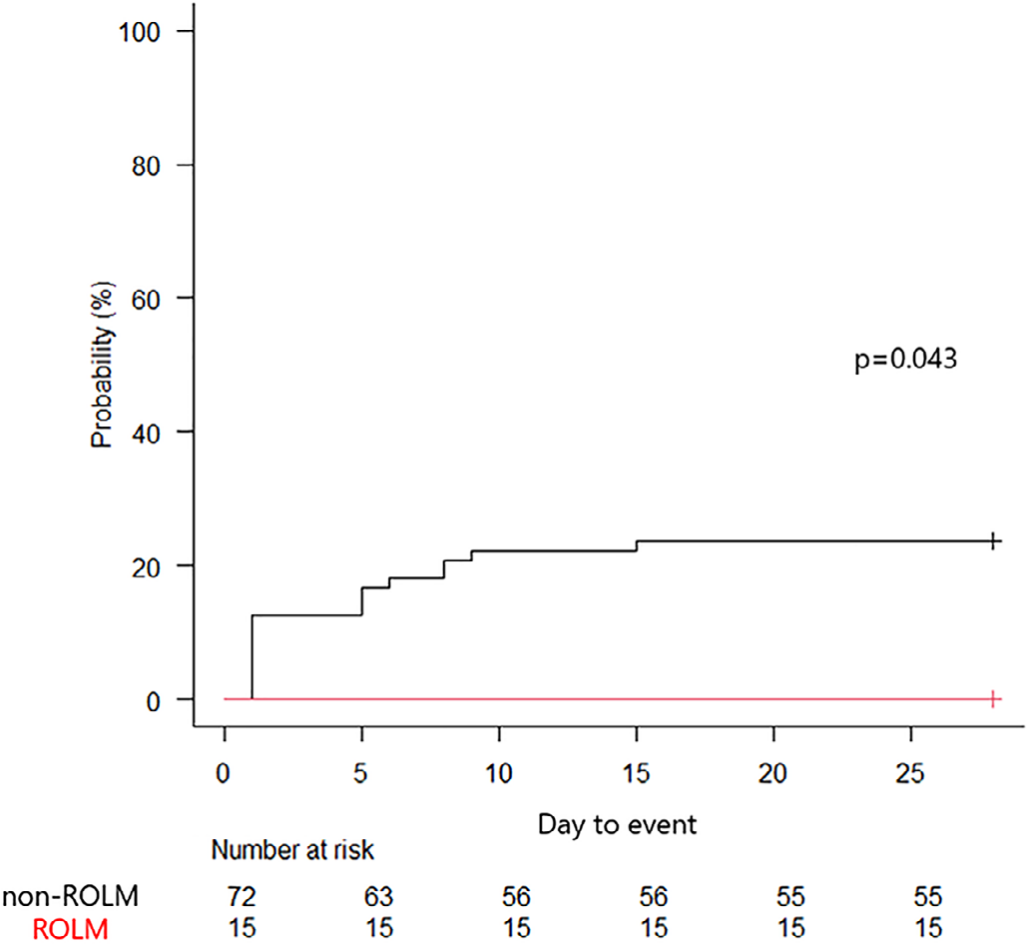
A Kaplan–Meier analysis.

Delayed bleeding occurred in 0/15 patients (0%) of the ROLM group and 17/72 patients (23.6%) of the non‐ROLM group, with a statistically significant difference (*p* = 0.036). Delayed perforation occurred in 0/15 patients (0%) of the ROLM group and 1/72 patients (1.4%) of the non‐ROLM group, with no significant difference. Kaplan–Meier analysis revealed a marked divergence in delayed bleeding over 28 days, with no events in the ROLM group and a rising cumulative incidence in the non‐ROLM group (*p* = 0.043, Figure 3).

**FIGURE 4:**
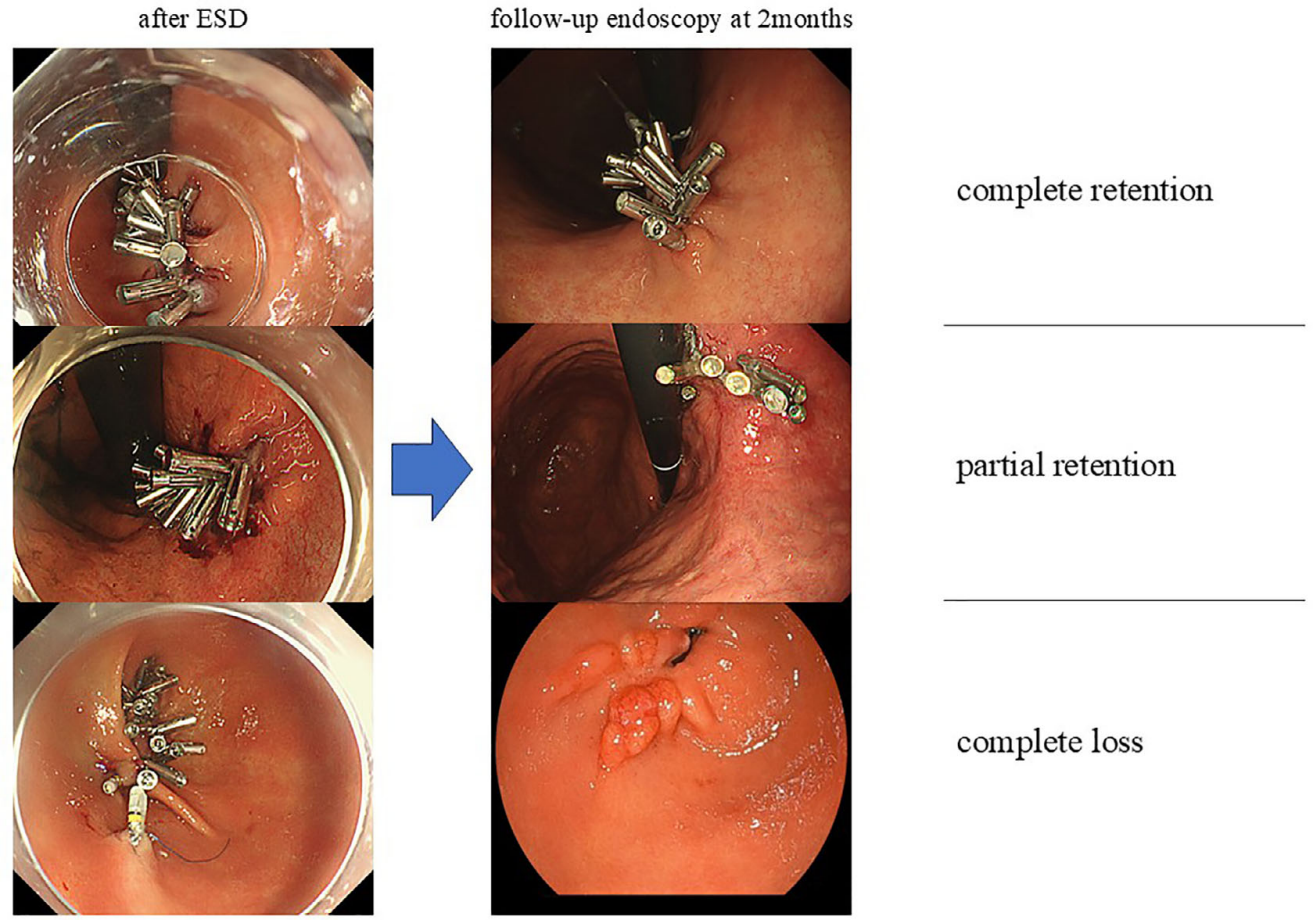
Classification of clip retention.

Sustained closure on POD1 was 21/21 lesions (100%) in the ROLM group. At 2 months, complete clip retention was observed in 4/21 lesions (19.0%), partial retention in 13/21 lesions (61.9%), and complete loss in 4/21 lesions (19.0%, Figure 4).

### 3.4 Risk Factor Analysis for Delayed Bleeding

Univariate analysis was performed to explore factors potentially associated with delayed bleeding (Table 4). The absence of ROLM was significantly associated with a higher incidence of delayed bleeding (0/15 [0%] vs. 17/72 [23.6%], *p* = 0.036), supporting its potential protective role. In addition, a CCI ≥2 was also significantly more common in the bleeding group compared to the non‐bleeding group (16/17 [94.1%] vs. 48/70 [68.6%], *p* = 0.035).

**TABLE 4:** Univariate analysis of factors associated with delayed bleeding.

	Bleeding *n* = 17	No bleeding *n* = 70	*p*
Sex (male/female)	17/0	59/11	1.000
Age, median (range), years	77 (58–89)	77 (61–88)	0.581
ECOG‐PS (0–1/2+)	17/0	65/5	0.578
ASA (1–2/3+)	4/13	30/40	0.174
CCI (0–1/2+)	1/16	22/48	0.035
Antiplatelet (yes/no)	12/5	35/35	0.176
Anticoagulant (yes/no)	5/12	36/34	0.115
HD (yes/no)	2/15	7/63	1.000
Multiple lesions (yes/no)	3/14	24/46	0.248
BEST‐J (3–4/5+)	13/4	53/17	1.000
Atrophy (closed/open/none)	3/14/0	10/58/2	0.818
ROLM (yes/no)	0/17	15/55	0.036

Abbreviations: ASA, American Society of Anesthesiologists physical status; CCI, Charlson Comorbidity Index; ECOG‐PS, Eastern Cooperative Oncology Group performance status; HD, hemodialysis; ROLM, reopenable clip‐over‐the‐line method.

Higher ASA physical status (≥3) and use of antiplatelet or anticoagulant agents also tended to be more frequent in the bleeding group, although these differences were not statistically significant.

Table 5 shows the multivariable analysis of the association between ROLM and delayed bleeding using the Firth logistic regression analysis. Although the unadjusted analysis showed a low odds ratio (OR) for ROLM (OR = 0.102), no statistical significance was reached (*p* = 0.131). To verify the robustness of the association, potential confounding factors (CCI, use of antithrombotic agents, hemodialysis, and multiple lesions) were identified based on univariate analysis. Multivariable Firth logistic regression models were subsequently constructed by sequentially incorporating these factors according to their medical relevance to evaluate the adjusted association between ROLM and delayed bleeding. In all adjusted models (Models a–d), ROLM consistently demonstrated point estimates of OR <0.1, indicating a stable trend toward a reduced risk of delayed bleeding. Although statistical significance was not achieved due to the limited number of bleeding events, consistent directionality across models supports the potential protective role of ROLM in the prevention of delayed bleeding.

**TABLE 5:** Multivariable analysis of the association between the reopenable clip‐over‐the‐line method (ROLM) and delayed bleeding.

	OR	95% CI	*p*
Unadjusted	0.102	0.005–1.972	0.131
Model^a^	0.087	0.004–1.675	0.106
Model^b^	0.080	0.005–1.402	0.084
Model^c^	0.082	0.005–1.378	0.082
Model^d^	0.087	0.005–1.449	0.089

Abbreviations: ROLM, reopenable clip‐over‐the‐line method.

^a^ Adjusted for Charlson Comorbidity Index (0–1 vs. 2+).

^b^ Adjusted for model a, antiplatelet and anticoagulant agents.

^c^ Adjusted for model b, and hemodialysis.

^d^ Adjusted for model c, and multiple lesions.

### 3.5 Factors Associated With Clip Retention

When comparing lesions with clip retention (*n* = 17) and those with complete clip loss (*n* = 4), no statistically significant differences were found in locations, specimen size, ROLM procedure time, or the number of clips used with ROLM. Although not statistically significant, lesions with complete clip loss tended to be located more frequently in the lower third of the stomach (Table 6).

**TABLE 6:** Factors associated with clip retention.

Factors	Retention present (*n* = 17)	Complete loss (*n* = 4)	*p*
Location 1 (middle/lower)	7/10	1/3	1.000
Location 2 (lesser curvature/greater curvature/anterior wall/posterior wall)	6/2/6/3	2/0/1/1	1.000
Specimen size, median (range), mm	35 (24–60)	30 (25–63)	0.393
ROLM procedure time, median (range), min	38 (18–74)	39.5 (22–73)	0.560
Number of clips used, median(range)	21 (13–36)	23 (12–39)	0.753

Abbreviations: ROLM, reopenable clip‐over‐the‐line method.

## 4 Discussion

This retrospective study showed no delayed bleeding in the ROLM group and 100% sustained mucosal closure on POD1. These outcomes highlight the potential of ROLM as a valuable prophylactic strategy in patients at high risk of post‐ESD bleeding.

Traditionally, post‐ESD ulcers without endoscopic closure have been managed conservatively with proton pump inhibitor (PPI) therapy alone, and complete healing typically requires up to 8 weeks [21].

Subsequently, vonoprazan, a novel potassium‐competitive acid blocker with stronger and faster acid suppression than conventional PPIs, was reported to reduce delayed bleeding after ESD [22]. However, its efficacy appears limited in patients receiving antithrombotic therapy [23], underscoring the need for adjunctive mechanical strategies to achieve effective hemostasis.

Mechanical closure techniques such as endoscopic hand suturing have shown promising preventive effects for delayed bleeding, even in patients receiving antithrombotic therapy [24].

More recently, Sugimoto et al. reported that ROLM significantly reduced delayed bleeding in a multicenter, propensity score‐matched cohort of gastric ESD cases, including a subset of high‐risk patients with BEST‐J ≥3 [15]. Although their study demonstrated the clinical efficacy of ROLM, it did not include a systematic evaluation of POD1 closure status or assessment of closure durability over time. In contrast, our routine POD1 second‐look endoscopy and scheduled 2‐month follow‐up allow direct assessment of early closure success and subsequent persistence of closure‐related structures. Therefore, our study complements prior work by providing additional insight into closure durability despite the small sample size.

In the present study, all ROLM lesions showed sustained closure on POD1, confirming immediate technical success. Furthermore, more than 80% of lesions exhibited clip retention at 2 months. Retained clips should not be interpreted as direct evidence of persistent mechanical closure because ROLM achieves mucosal apposition mainly through the line tension rather than the clips themselves. However, as the mucosal configuration becomes difficult to assess over time due to ulcer healing, clip persistence provides a practical and exploratory surrogate for the durability of the closure construct. Prolonged coverage may have contributed to protecting the ulcer‐bed and exposed vessels from gastric contents. This finding may provide a plausible mechanistic explanation for the prevention of delayed bleeding.

In this study, complete clip loss at 2 months occurred in four cases, three of which were located in the lower third of the stomach, suggesting that location may influence clip retention durability. Sugimoto et al. reported that all bleeding cases in the ROLM group were located in the antrum [15]. This may reflect increased mechanical stress due to antral motility, which may have contributed to earlier clip dislodgement [25]. Although the precise timing of clip loss in this study remains unknown, none of the 4 cases with complete clip loss experienced delayed bleeding.

Regarding the mucosal protection period needed to prevent delayed bleeding, our findings provide additional insight. In the non‐ROLM group, one patient experienced delayed bleeding around day 15, but no further bleeding events occurred after 3 weeks. Similarly, although conducted in standard‐risk patients, the study by Kato et al. reported no delayed bleeding after 3 weeks of vonoprazan administration [26]. Based on these findings, it is plausible that maintaining mucosal closure for approximately 3 weeks may be sufficient to reduce the risk of delayed bleeding. Further studies are needed to validate the optimal closure duration for effective hemostatic protection.

Despite these promising findings, this study had some limitations. First, the sample size was relatively small, particularly in the ROLM group. Although none of the Firth logistic regression models reached statistical significance, they consistently demonstrated a trend toward reduced bleeding risk with ROLM. This lack of significance is likely attributable to the limited number of patients in the ROLM group, which may have reduced the statistical power of the analysis. Second, this was a single‐center retrospective study. Although we attempted to mitigate selection bias and baseline imbalances using propensity score‐based IPTW, residual confounding factors inherent to the retrospective design cannot be completely excluded. Finally, comparisons with other closure techniques, such as loop clip methods or suturing devices, were not included in this study, which remain an important topic for future research.

Our findings suggest that ROLM is a feasible, durable, and low‐barrier technique that may prevent delayed bleeding after gastric ESD, particularly in high‐risk patients. Larger prospective comparative studies are warranted to confirm these results and further define the role of ROLM in routine clinical practice.

## 5 Conclusion

ROLM is an effective method of closing mucosal defects after gastric ESD in high‐risk patients. Sustained closure was achieved in all cases on POD1, and clip retention was observed in over 80% at 2 months. Delayed bleeding did not occur in the ROLM group, suggesting that this technique may help prevent post‐ESD bleeding. However, further prospective studies are required to confirm these results.

## Author Contributions

All authors contributed to the conception and design of the study. **Gen Kitahara** performed the data collection. **Gen Kitahara**, **Takuya Wada**, and **Kenji Ishido** reviewed POD1 and the 2‐month follow‐up assessment. **Kenta Murotani** designed the statistical analysis and conducted the analysis. **Gen Kitahara** drafted the initial manuscript, and all authors revised it critically for intellectual content. All authors approved the final version of the manuscript and agree to be accountable for all aspects of the work, ensuring the accuracy and integrity of the study.

## Conflicts of Interest

The authors declare no conflicts of interest.

## Funding Information

The authors received no specific funding for this work.

## Ethics Statement

The current study adhered to the principles outlined in the Declaration of Helsinki and was approved by the Ethics Committee of Kitasato University School of Medicine Hospital (B24‐003).

## Consent

Informed consent (opt‐out) was obtained from all patients in compliance with the Japanese Guidelines for Human Medical Research.

## Clinical Trial Registration

N/A

## Supporting information




**Table S1**: Patient characteristics after inverse probability of treatment weighting (IPTW).

## References

[1] P. W. Chiu , A. Y. B. Teoh , K. F. To , et al., “Endoscopic Submucosal Dissection (ESD) Compared With Gastrectomy for Treatment of Early Gastric Neoplasia: A Retrospective Cohort Study,” Surgical Endoscopy 26 (2012): 3584–3591. 22678176 10.1007/s00464-012-2371-8

[2] H. Ono , N. Hasuike , T. Inui , et al., “Usefulness of a Novel Electrosurgical Knife, the Insulation‐tipped Diathermic Knife‐2, for Endoscopic Submucosal Dissection of Early Gastric Cancer,” Gastric Cancer 11 (2008): 47–52. 18373177 10.1007/s10120-008-0452-0

[3] S. Oka , S. Tanaka , I. Kaneko , et al., “Advantage of Endoscopic Submucosal Dissection Compared With EMR for Early Gastric Cancer,” Gastrointestinal Endoscopy 64 (2006): 877–883. 17140890 10.1016/j.gie.2006.03.932

[4] J. Lian , S. Chen , Y. Zhang , and F. Qiu , “A Meta‐analysis of Endoscopic Submucosal Dissection and EMR for Early Gastric Cancer,” Gastrointestinal Endoscopy 76 (2012): 763–770. 22884100 10.1016/j.gie.2012.06.014

[5] I. Oda , H. Suzuki , S. Nonaka , and S. Yoshinaga , “Complications of Gastric Endoscopic Submucosal Dissection,” Digestive Endoscopy 25, no. suppl. 1 (2013): 71–78. 10.1111/j.1443-1661.2012.01376.x 23368986

[6] S. Ono , M. Ono , M. Nakagawa , Y. Shimizu , M. Kato , and N. Sakamoto , “Delayed Bleeding and Hemorrhage of Mucosal Defects After Gastric Endoscopic Submucosal Dissection on Second‐look Endoscopy,” Gastric Cancer 19 (2016): 561–567. 26089283 10.1007/s10120-015-0507-y

[7] T. Yano , S. Tanabe , K. Ishido , et al., “Different Clinical Characteristics Associated With Acute Bleeding and Delayed Bleeding After Endoscopic Submucosal Dissection in Patients With Early Gastric Cancer,” Surgical Endoscopy 31 (2017): 4542–4550. 28378078 10.1007/s00464-017-5513-1

[8] H. Suzuki , K. Takizawa , T. Hirasawa , et al., “Short‐term Outcomes of Multicenter Prospective Cohort Study of Gastric Endoscopic Resection: ‘Real‐world Evidence’ in Japan,” Digestive Endoscopy 31 (2019): 30–39. 30058258 10.1111/den.13246

[9] W. Hatta , Y. Tsuji , T. Yoshio , et al., “Prediction Model of Bleeding After Endoscopic Submucosal Dissection for Early Gastric Cancer: BEST‐J Score,” Gut 70 (2021): 476–484. 32499390 10.1136/gutjnl-2019-319926 PMC7873424

[10] M. Ego , S. Abe , S. Nonaka , et al., “Endoscopic Closure Utilizing Endoloop and Endoclips After Gastric Endoscopic Submucosal Dissection for Patients on Antithrombotic Therapy,” Digestive Diseases and Sciences 66 (2021): 2336–2344. 32797345 10.1007/s10620-020-06508-8

[11] Y. Yamasaki , Y. Takeuchi , M. Kato , N. Uedo , and R. Ishihara , “Line‐assisted Complete Closure of Large Gastric Mucosal Defects by Use of Multiple Clip‐and‐line Technique,” VideoGIE 1 (2016): 49–50. 29905240 10.1016/j.vgie.2016.08.008 PMC5989482

[12] N. Nishiyama , H. Kobara , N. Kobayashi , et al., “Novel Endoscopic Ligation With O‐ring Closure Involving Muscle Layer of a Gastric Artificial Defect,” Endoscopy 52 (2020): E413–E414. 32330951 10.1055/a-1149-8597

[13] O. Goto , T. Oyama , H. Ono , et al., “Endoscopic Hand‐suturing Is Feasible, Safe, and May Reduce Bleeding Risk After Gastric Endoscopic Submucosal Dissection: A Multicenter Pilot Study (With video),” Gastrointestinal Endoscopy 91 (2020): 1195–1202. 31923410 10.1016/j.gie.2019.12.046

[14] T. Nomura , S. Sugimoto , T. Temma , J. Oyamada , and A. Kamei , “Clip‐line Closure With the Reopenable Clip Over Line Method for a Large Mucosal Defect After Gastric Endoscopic Submucosal Dissection,” Endoscopy 54 (2022): E1–E2. 33592649 10.1055/a-1346-8991

[15] S. Sugimoto , T. Nomura , T. Temma , et al., “Closure of Gastric Mucosal Defects Using the Reopenable‐clip Over‐the‐line Method to Decrease the Risk of Bleeding After Endoscopic Submucosal Dissection: A Multicenter Propensity Score‐matched Case‐control Study (With video),” Gastrointestinal Endoscopy 102 (2025): 37–46. 39542223 10.1016/j.gie.2024.11.015

[16] American Society of Anesthesiologists . ASA Physical Status Classification System . [Cited October 2, 2024]., https://www.asahq.org/resources/clinical‐information/asaphysicalstatus/classification/system.

[17] M. E. Charlson , P. Pompei , K. L. Ales , and C. R. MacKenzie , “A New Method of Classifying Prognostic Comorbidity in Longitudinal Studies: Development and Validation,” Journal of Chronic Diseases 40 (1987): 373–383. 3558716 10.1016/0021-9681(87)90171-8

[18] M. Kato , N. Uedo , S. Hokimoto , et al., “Guidelines for Gastroenterological Endoscopy in Patients Undergoing Antithrombotic Treatment: 2017 Appendix on Anticoagulants Including Direct Oral Anticoagulants,” Digestive Endoscopy 30 (2018): 433–440. 29733468 10.1111/den.13184

[19] Japanese Gastric Cancer Association . Japanese Gastric Cancer Treatment Guidelines 2021 (6th Edn). Gastric Cancer 26 (2023): 1–25. 36342574 10.1007/s10120-022-01331-8 PMC9813208

[20] R. Puhr , G. Heinze , M. Nold , L. Lusa , and A. Geroldinger , “Firth's Logistic Regression With Rare Events: Accurate Effect Estimates and Predictions?” Statistics in Medicine 36 (2017): 2302–2317. 28295456 10.1002/sim.7273

[21] N. Kakushima , M. Fujishiro , N. Yahagi , S. Kodashima , M. Nakamura , and M. Omata , “Helicobacter pylori Status and the Extent of Gastric Atrophy Do Not Affect Ulcer Healing After Endoscopic Submucosal Dissection,” Journal of Gastroenterology and Hepatology 21 (2006): 1586–1589. 16928221 10.1111/j.1440-1746.2006.04321.x

[22] K. Hamada , N. Uedo , Y. Tonai , et al., “Efficacy of vonoprazan in Prevention of Bleeding From Endoscopic Submucosal Dissection‐induced Gastric Ulcers: A Prospective Randomized Phase II Study,” Journal of Gastroenterology 54 (2019): 122–130. 29943163 10.1007/s00535-018-1487-6

[23] H. Abe , K. Tarasawa , W. Hatta , et al., “Which of vonoprazan Alone or Intravenous Proton Pump Inhibitor Followed by vonoprazan Is Optimal for Reducing Delayed Bleeding in Gastric Endoscopic Submucosal Dissection?” Digestion 106, no. 5 (2025): 406–415. 40132569 10.1159/000545253 PMC12060810

[24] T. Akimoto , O. Goto , M. Sasaki , et al., “Endoscopic Hand Suturing for Mucosal Defect Closure After Gastric Endoscopic Submucosal Dissection May Reduce the Risk of Postoperative Bleeding in Patients Receiving Antithrombotic Therapy,” Digestive Endoscopy 34 (2022): 123–132. 34021512 10.1111/den.14045

[25] K. Jones , M. Edelbroek , M. Horowitz , et al., “Evaluation of Antral Motility in Humans Using Manometry and Scintigraphy,” Gut 37 (1995): 643–648. 8549939 10.1136/gut.37.5.643 PMC1382868

[26] M. Kato , N. Hosoe , T. Gotoda , et al., “Treatment With Vonoprazan for 3 Weeks Is Not Inferior to 8 Weeks for the Management of Gastric ESD: A Multicenter Noninferiority Randomized Study,” Journal of Gastroenterology 58 (2023): 358–366. 36781490 10.1007/s00535-023-01966-z

