# Supplementary material for: Evaluation of Delayed Bleeding Prevention and Sustained Closure Using the Reopenable Clip‐Over‐the‐Line Method for Gastric Endoscopic Submucosal Dissection
Source: DEN Open. 2026 Feb 26;6(1):e70302. doi: 10.1002/deo2.70302 (PMC12938493; doi:10.1002/deo2.70302)
Supplement: Supplementary file 1 — Table S1: Patient characteristics after inverse probability of treatment weighting (IPTW). [file DEO2-6-e70302-s001.docx]

Supplementary Table 1.

Patient characteristics after IPTW (delayed bleeding shown for reference)

Supplementary Table 1.

|  | ROLM　n=11.2 | non-ROLM　n=75.9 | p | SMD |
| --- | --- | --- | --- | --- |
| Sex (Male/Female) | 10.1/1.1 | 67.2/8.7 | 0.852 | 0.054 |
| Age, median(range), years | 77.3(64-87) | 78.0(58-89) | 0.952 | 0.063 |
| ECOG-PS (0-1/2+) | 9.8/1.4 | 67.6/8.2 | 0.887 | 0.053 |
| ASA (1-2/3+) | 3.4/7.8 | 27.4/48.5 | 0.696 | 0.120 |
| CCI (0-1/2+) | 1.7/9.5 | 18.6/57.3 | 0.408 | 0.244 |
| Antiplatelet (yes/no) | 4.3/6.9 | 32.7/43.1 | 0.783 | 0.101 |
| Anticoagulant (yes/no) | 5.6/5.6 | 42.1/33.8 | 0.760 | 0.105 |
| HD (yes/no) | 9.1/2.0 | 63.6/12.2 | 0.879 | 0.051 |
| Multiple lesions (yes/no) | 7.8/3.3 | 54.1/21.7 | 0.935 | 0.025 |
| BEST-J (3-4/5+) | 5.4/5.7 | 53.8/22.1 | 0.186 | 0.465 |
| Atrophy (closed/open/none) | 0.4/10.5/0.3 | 10.4/64.2/1.3 | 0.325 | 0.383 |
| Delayed bleeding (yes/no) * | 0/11.2 | 18.1/57.7 | 0.094 | N/A |

ROLM, reopenable clip-over-the-line method; SMD, standardized mean differences

ECOG-PS, Eastern Cooperative Oncology Group performance status; ASA, American Society of Anesthesiologists physical status; CCI, Charlson Comorbidity Index; HD, hemodialysis

*Delayed bleeding is shown for reference only and was not used for covariate balance assessment
